# Evaluation of the Effectiveness of Endoscopic Retrograde Cholangiopancreatography in Patients with Perihilar Cholangiocarcinoma and Its Effect on Development of Cholangitis

**DOI:** 10.1155/2014/508286

**Published:** 2014-05-27

**Authors:** Serkan İpek, Emrah Alper, Cem Cekic, Serkan Cerrah, Mahmut Arabul, Fatih Aslan, Belkis Unsal

**Affiliations:** ^1^Department of Gastroenterology, Atatürk Training and Research Hospital, İzmir Katip Çelebi University, İzmir Atatürk Eğitim ve Araştırma Hastanesi, Karabağlar, 35160 İzmir, Turkey; ^2^Department of Gastroenterology, Erzurum Regional and Research Hospital, 25140 Erzurum, Turkey

## Abstract

*Objective.* We aimed to determine the effectiveness of endoscopic retrograde cholangiopancreatography (ERCP) in patients with inoperable perihilar cholangiocarcinoma and establish the incidence of cholangitis development following ERCP. *Material and Method.* This retrospective study enrolled patients diagnosed with inoperable perihilar cholangiocarcinoma who underwent endoscopic drainage (stenting) with ERCP. Patients were evaluated for development of cholangitis and the effectiveness of ERCP. The procedure was considered successful if bilirubin level fell more than 50% within 7 days after ERCP. *Results.* Post-ERCP cholangitis developed in 40.7% of patients. Cholangitis development was observed among 39.4% of patients with effective ERCP and in 60.6% of patients with ineffective ERCP. Development of cholangitis was significantly more common in the group with ineffective ERCP compared to the effective ERCP group (*P* = 0.001). The average number of ERCP procedures was 2.33 ± 0.89 among patients developing cholangitis and 1.79 ± 0.97 in patients without cholangitis. The number of ERCP procedures was found to be significantly higher among patients developing cholangitis compared to those without cholangitis (*P* = 0.012). *Conclusion.* ERCP may not provide adequate biliary drainage in some of the patients with perihilar cholangiocarcinoma and also it is a procedure associated an increased risk of cholangitis.

## 1. Introduction

Cholangiocarcinomas which include intrahepatic and extrahepatic forms represent the most common primary malignancy of the biliary tract. Extrahepatic cholangiocarcinoma is further divided into two groups, perihilar and distal extrahepatic cholangiocarcinoma, at the cystic duct level. Perihilar cholangiocarcinoma, also known as Klatskin tumor, is the most common type of cholangiocarcinoma. Histopathologically, it is mostly a moderate-to-well differentiated, mucin-producing adenocarcinoma with abundant fibrous stroma. Prognosis of cholangiocarcinoma is poor due to its silent clinical character, the difficulty of early diagnosis, and limited therapeutic approaches. It has an average survival of less than 24 months [1]. Clinical presentation depends on the localization, stage, and growth pattern of the tumor. Clinically, 90% of patients with perihilar cholangiocarcinoma present with biliary problems including painless jaundice, whereas 10% present with cholangitis [2]. While the primary treatment of perihilar cholangiocarcinoma is surgical resection with or without hepatectomy, chances for curative therapy are considerably low because of delayed diagnosis (20–40%) [3]. For inoperable patients, endoscopic retrograde cholangiopancreatography (ERCP) or percutaneous transhepatic biliary drainage is considered in order to alleviate symptoms and prevent complications related to chronic cholestasis [4].

In the present study, we aimed to determine the effectiveness of endoscopic retrograde cholangiopancreatography (ERCP) for symptomatic treatment (icterus) in patients with inoperable perihilar cholangiocarcinoma and establish the incidence of cholangitis development following ERCP.

## 2. Material and Method

The study was conducted by retrospective screening of patients undergoing ERCP at ERCP Unit of Gastroenterology Clinic in Izmir Katip Celebi University, Ataturk Research and Training Hospital, between January 2009 and January 2012. Enrolled patients were those who had been previously diagnosed with perihilar cholangiocarcinoma using cytologic sampling by ERCP or EUS and considered to have an inoperable tumor on the basis of results of advanced imaging techniques (dynamic MRI, dynamic CT) and undergone endoscopic drainage (stenting) by ERCP. Patients were evaluated in terms of clinical signs and laboratuary values before ERCP. Cholangitis cases were excluded. Therefore, the bile samples for culture before stent placement were not taken in patients who were included in the study. Patients were divided into subgroups according to Bismuth-Corlette classification.

ERCP had been performed after 12-hour fasting using a standard therapeutic duodenoscope (Olympus Duodenoscope TJF-Q150) under sedative anesthesia induced by dormicum-propofol. Choledochus had been cannulated by a standard sphincterotome (Boston Scientific Ultratome XL) using a guidewire. After confirming the entry of guidewire in the choledochus under the scope, choledochus had been stained using minimal amount of iodinated contrast agent (Omnipaque), taking care to allow as low penetration of contrast material as possible to the proximal of stenosis. Subsequent to obtaining cytologic specimens from the stenosed site using a brush, a 7-, 8.5-, or 10-FR plastic stent had been inserted depending on the degree of stenosis. We performed dilatation with 8 mm balloon for broader stenting in eight patients. For all patients, upper edge of the stent was placed in the proximal of the stenosis and bile flow was observed during the procedure.

White blood cell (WBC) count, hematocrit (Hct), bilirubin, alanine aminotransferase (ALT), alkaline phosphatase (ALP), C-reactive protein (CRP), and fever had been monitored for all patients before (12–24 hours) and after ERCP (1–10 days). Hemocultures had been obtained if body temperature exceeded 37.8°C on at least three consecutive occasions within 1 hour. WBC and CRP had been followed daily for patients with elevated temperatures and with 2-day intervals for other patients. Empirical treatment with third-generation cephalosporins had been started in patients who had leukocytosis and hemoculture in addition to high fever. Changes in antibiotic therapy had been made for patients with a positive culture result according to the antibiogram.

The procedure was considered successful if bilirubin level fell more than 50% within 7 days after ERCP. Newly developed post-ERCP cholangitis was considered if the body temperature of the patient exceeded 37.8°C, WBC was >9000/*μ*L or <4000/*μ*L, and CRP levels were increased within 7 days of ERCP.

SPSS 17.0 software package was used for statistical analysis. Categorical measurements were summarized as number and percentage and continuous measurements as mean and standard deviations. Chi-square test was used for comparing categorical variables. Student's *t* test was used for comparing continuous variables between groups. Statistical significance was set at 0.05 for all tests.

The study began after obtaining approval from the local ethics committee.

## 3. Results

Results of 81 patients were reviewed; all of these patients had been diagnosed with perihilar cholangiocarcinoma using cytologic sampling (ERCP or EUS) and considered to have an inoperable tumor on the basis of results of advanced imaging studies (dynamic MRI, dynamic CT) and undergone endoscopic drainage (stenting) by ERCP. Of study patients, 40 were females (49.4%) and 41 (50.6%) were males, with a mean age of 71.09 ± 11.98 years. Para-aortic and celiac lymph node involvement in 58 of the patients and liver metastases in 23 patients were present. The average number of ERCP procedures was 2.01 ± 0.97. More than 50% decrease in bilirubin level within 7 days, which was considered to indicate the effectiveness of ERCP or success of the procedure, was found in 49 (60.5%) patients. ERCP was found to be ineffective among 32 patients (39.5%). Thirty-three patients (40.7%) had developed cholangitis after the procedure. Cholangitis development was observed among 60.6% of patients with inadequate drainage during ERCP and in 39.4% of patients with effective ERCP. Development of cholangitis was significantly more common in the group with ineffective ERCP compared to the effective ERCP group (*P* = 0.001). There was no significant difference between groups with effective or ineffective ERCP with respect to gender (*P* = 0.413). When patients were evaluated on the basis of Bismuth-Corlette classification; ERCP was observed to be effective in 12 out of 20 patients with type 1 tumor (60%), 31 out of 50 patients with type 2 tumor (62%), and 6 out of 9 patients with type 3 tumor (66.7%). ERCP was found ineffective in both of two patients with type 4 tumor (Table 1).

The average number of ERCP procedures was 2.33 ± 0.89 among patients developing cholangitis and 1.79 ± 0.97 for patients without cholangitis. The number of ERCP procedures was found to be significantly higher among patients developing cholangitis compared to those without cholangitis (*P* = 0.012). While the average ERCP number was 2.04 ± 0.98 in patients with effective ERCP, it was 1.97 ± 0.97 for patients with ineffective ERCP. Although the number of ERCP procedures performed was higher in the group of patients in whom ERCP was effective compared to the group with ineffective ERCP, no significant difference was found between them (*P* = 0.746) (Table 2).

When patients were divided into subgroups according to Bismuth classification, it was found that 7 out of 20 patients with type 1 tumor (35%), 20 out of 50 patients with type 2 tumor (40%), 4 out of 9 patients with type 3 tumor (44%), and both of 2 patients with type 4 tumor (100%) had developed post-ERCP cholangitis (Table 3).

## 4. Discussion

Perihilar cholangiocarcinoma is usually diagnosed in the later stages of the disease due to absence of early symptoms. The major clinical symptom is jaundice [5]. Only 10%–20% of all patients are candidates for curative therapy. Thus, only palliative drainage therapy can be given to the majority of patients [3]. The goal of palliative treatment is to achieve improved quality of life [6].

Currently, there is no consensus on the best method for palliation of jaundice among patients with unresectable Klatskin tumors [7]. Successful biliary drainage following endoscopic stent placement or percutaneous transhepatic endoprosthesis may provide some benefits for palliation of jaundice and improve the quality of life in patients with unresectable Klatskin tumors [8]. Similarly, the same methods can be used preoperatively for operable tumors but there is still controversy over which method should be preferred. Percutaneous transhepatic biliary drainage is preferred mainly in Japan [9, 10]. However, ERCP is generally used as the primary intervention in Europe and USA. If ERCP is inadequate, it is followed by percutaneous methods. Previous studies have reported that although ERCP is less invasive, it is associated with an increased risk for development of cholangitis due to bacterial contamination from duodenum [11].

Previously, success rates ranging between 41% and 88% have been reported with use of ERCP for palliation of cholestasis [12–16]. In our study, successful biliary drainage was achieved in 60.5% of patients with perihilar cholangiocarcinoma who had undergone ERCP. In the remaining 39.5% of patients, a sufficient decrease in bilirubin level could not be achieved, showing failure of the drainage. When patients were evaluated on the basis of Bismuth-Corlette classification, ERCP was found to be effective in 60% of patients with type 1 tumor, 62% of patients with type 2 tumor, and 66.7% of patients with type 3 tumor. However, ERCP was ineffective in both of two patients with type 4 tumor. While some studies [7, 13] have reported that the success rate of biliary drainage by ERCP was lower in types 3 and 4 patients versus types 1 and 2 patients, in the present study we found a higher success rate (albeit nonsignificant) among type 3 patients compared to type 1 or type 2. This might have resulted from the small number of type 3 patients.

Post-ERCP cholangitis developed in 40.7% of patients. Cholangitis development was observed among 39.4% of patients with effective ERCP and in 60.6% of patients with ineffective ERCP. Development of cholangitis was significantly more common in the group with ineffective ERCP compared to the effective ERCP group (*P* = 0.001).

Previous studies in literature have reported an incidence of cholangitis varying between 3.4% and 5.8% for all ERCP procedures combined. Contrastingly, post-ERCP cholangitis is present in 6%–49% of patients with Klatskin tumor. This condition is observed particularly among patients in whom drainage of biliary segments following injection of contrast agent fails [17]. This piece of information might explain why the rate of cholangitis development was significantly higher in the group with ineffective ERCP in our study. For ERCP procedures which fail to provide adequate bilirubin reduction, resulting in insufficient drainage, the risk for cholangitis may be expected to rise when contrast material is concomitantly injected. We used as little amount of iodinated contrast agent as possible for imaging studies in order to minimize that risk. We gave the contrast agent in such an amount that would suffice to get images only from distal part of the tumor but would not reach the proximal of the tumor for patients with a clear vision of stenosis as conferred by USG and MRI cholangiography.

Lee et al. reported a success rate of 79.4% for palliation of cholestasis among ERCP patients in their study which evaluated optimal biliary drainage in patients with Klatskin tumor. In that study, they also evaluated patients who have undergone external percutaneous transhepatic biliary drainage (EPTBD) and internal biliary stenting via the percutaneous transhepatic biliary drainage tract (IPTBD) and found a higher success rate of palliation in both EPTBD (93.9%) and IPTBD (97.1%) groups compared to ERCP. Additionally, cholangitis development was more common with ERCP (29.4%) or IBTBD (32.4%) compared to EBTBD (12.1%). In the same study, the success rate was similar for Bismuth type II and lower for Bismuth type III and type IV when ERCP patients were compared with other groups [7]. In a study by Born et al. comparable rates of cholangitis were shown versus ERCP [18].

In their study, Rerknimitr et al. divided patients into three groups as group 1 = Bismuth I, group 2 = Bismuth II and group 3 = Bismuth III and IV. Post-ERCP cholangitis rates were 4% in group 1, 10% in group 2, and 57.7% in group 3. Resolution of jaundice was detected in 96% of group 1, 100% of group 2, and 73.1% in group 3. As a result, they reported that outcome of biliary drainage was poorer among patients with Bismuth III and IV tumors compared to those with Bismuth I and II tumors [13].

Walter et al. reported a success rate of 68% for the endoscopic biliary drainage (EBD) group and 98% for percutaneous transhepatic biliary drainage (PTBD) group. Cholangitis rates were higher in the EBD group versus the PTBD group [19]. Silva et al. reported an endoscopic success rate of 20% and stated that 80% of patients needed percutaneous biliary drainage [20]. Nimura reported that preoperative biliary drainage and, particularly, EBD increased the risk for cholangitis in patients with proximal cholangiocarcinoma [9]. Liu et al.'s study found that ERCP was associated with a success rate of 41%. They observed that 87.5% of patients with inadequate biliary drainage despite the fact that stenting had type III or type IV obstruction. Cholangitis was detected in 22% of patients [12].

Arvanitakis et al. found a success rate of 65% in their study which investigated predictive factors for survival among patients with inoperable Klatskin tumors. They reported that baseline bilirubinemia was the sole factor predicting successful drainage. Reported predictive factors for 12-month survival included mass-type tumor or absence of hepatic metastasis, no related complications, and successful drainage [21].

Kloek et al. achieved successful drainage in 81% of patients by ERCP and 100% of patients by percutaneous drainage in their study, with no statistically significant difference between the two procedures. While 48% of ERCP patients developed cholangitis, the corresponding rate of cholangitis was 9% among patients undergoing percutaneous drainage. There were significantly more patients developing cholangitis in the group receiving ERCP [22].

Several comparative studies published since 2000 [19] have reported that percutaneous transhepatic biliary drainage was associated with a higher rate of therapeutic success versus endoscopic biliary drainage (71%–97% for PTBD, 41%–79% for EBD). Definition of therapeutic success rate varies between studies in literature. We defined therapeutic success as 50% decrease in bilirubin levels within the first week. Other research groups have defined therapeutic success as a more than 20% or 50% decrease in bilirubin levels or reduction of total bilirubin below 40 *μ*mol/L [19]. Thus, most of the studies have different definitions for a successful biliary drainage.

In many centers, endoscopic stenting is considered as the first-line treatment because it is less invasive compared to percutaneous approach. ERCP may lead to cholangitis due to a biliary segment failing to drain following injection of contrast agent. Thus, the most effective method for palliative biliary drainage may be identified on the basis of the level or type of biliary obstruction in patients with an unresectable Klatskin tumor [7]. Taking into account the results of some previous studies indicating a higher rate of cholangitis and achievement of lower biliary drainage rates among patients with Bismuth types III and IV tumors [7, 13], percutaneous drainage may be considered for these patients. In the present study, 35% of patients with type I tumor, 40% of patients with type II tumor, 44% of patients with type III tumor, and both of 2 patients with type 4 tumor (100%) had developed post-ERCP cholangitis. The small number of patients with Bismuth type IV tumor included in our study precluded adequate assessment of this issue.

In a study, Pisello et al. used air instead of iodinated contrast agent during ERCP for patients with Klatskin tumors. Cholangitis developed at a significantly lower rate in the air cholangiography group compared to the iodinated contrast group. They reported that use of air as contrast agent for obtaining cholangiography before placement of the stent might help prevent cholangitis particularly in patients with Klatskin tumors [17].

In conclusion, ERCP may not provide adequate biliary drainage in patients with perihilar cholangiocarcinoma and it is associated with an increased risk of cholangitis. Therefore, in patients with perihilar cholangiocarcinoma to provide adequate biliary drainage and to reduce the risk of cholangitis such as photodynamic therapy and radiofrequency ablation therapy for local tumor control should be considered [4, 23]. Based on previous studies, we suggest that it would be more appropriate to prefer percutaneous drainage for tumors with more proximal localization and to use air in lieu of contrast agent. However, further large-scale studies are needed to support these arguments.

## Figures and Tables

**Table 1:** Distribution of patients with respect to gender, development of cholangitis, and tumor type by ERCP effectiveness.

	Ineffective ERCP *n* (%)	Effective ERCP *n* (%)	*P*
Gender			
Male	18 (43.9)	23 (56.1)	0.413
Female	14 (35)	26 (65)	
Cholangitis			
No	12 (25)	36 (75)	0.001
Yes	20 (60.6)	13 (39.4)	
Tumor type			
I	8 (40)	12 (60)	—
II	19 (38)	31 (62)	
III	3 (33.3)	6 (66.7)	
IV	2 (100)	0 (0)	

Total	32 (39.5)	49 (60.5)	

**Table 2:** Effectiveness of ERCP and cholangitis development by ERCP number.

	*n* (%)	ERCP number (mean ± SD)	*P*
Effectiveness of ERCP			
Effective	49 (60.5)	2.04 ± 0.98	0.746
Ineffective	32 (39.5)	1.97 ± 0.97	
Cholangitis			
Yes	33 (40.7)	2.33 ± 0.89	0.012
No	48 (59.3)	1.79 ± 0.97	

**Table 3:** Distribution of patients with respect to gender and tumor type by cholangitis development.

	Patients with cholangitis *N* (%)	Patients without cholangitis *N* (%)	*P*
Gender			
Male	18 (43.9)	23 (56.1)	0.558
Female	15 (37.5)	25 (62.5)	
Tumor type			
I	7 (35)	13 (65)	—
II	20 (40)	30 (60)	
III	4 (44.4)	5 (55.6)	
IV	2 (100)	0 (0)	

Total	33 (40.7)	48 (59.3)	
